# Effects of technology-based exercise programs in older adults: A systematic review and meta-analysis

**DOI:** 10.1093/geroni/igaf122.4378

**Published:** 2025-12-31

**Authors:** Sunghee Tak, Hyein Choi, Sunjung Kim

**Affiliations:** Seoul National University, Seoul National Univerisity, Seoul-t’ukpyolsi, Republic of Korea; Seoul National University, Seoul National Univerisity, Seoul-t’ukpyolsi, Republic of Korea; Seoul National University, Seoul National Univerisity, Seoul-t’ukpyolsi, Republic of Korea

## Abstract

**Background:**

Exergame and virtual reality–based interventions have emerged as innovative strategies to prevent falls in older adults. However, evidence regarding their effects on key outcomes related to fall prevention remains limited.

**Methods:**

A systematic review and meta-analysis was conducted following PRISMA guidelines. Six electronic databases were searched to identify randomized controlled trials and quasi-experimental studies involving participants aged ≥60 years that reported at least one relevant outcome (mobility, balance, or fear of falling). Data were synthesized using a random-effects model, and standardized mean differences (SMDs) with 95% confidence intervals (CIs) were calculated. Risk of publication bias was assessed using funnel plots.

**Results:**

Nine studies met the inclusion criteria. Meta-analysis showed significant improvements in mobility (SMD = –1.05, 95% CI: –1.58 to –0.51) and dynamic balance measured by the Berg Balance Scale (SMD = 0.46, 95% CI: 0.10 to 0.83). No significant effect was found for static balance assessed by the One-Leg Stance test (SMD = –0.28, 95% CI: –1.00 to 0.43). Fear of falling was significantly reduced (SMD = –0.35, 95% CI: –0.61 to –0.08), but the effect became non-significant after excluding a study with median-based statistics (SMD = –0.021, 95% CI: –0.58 to 0.16). Funnel plots indicated possible publication bias for mobility and fear of falling.

**Conclusions:**

Technology-based interventions may improve mobility and dynamic balance in older adults, but their effects on static balance and fear of falling are less certain. High-quality, methodologically rigorous trials are needed to confirm these findings and guide their integration into geriatric fall-prevention programs.

